# Supplementary figures and images for: Rif2 protects Rap1-depleted telomeres from MRX-mediated degradation in Saccharomyces cerevisiae
Source: eLife. 2022 Jan 19;11:e74090. doi: 10.7554/eLife.74090 (PMC8791636; doi:10.7554/eLife.74090)

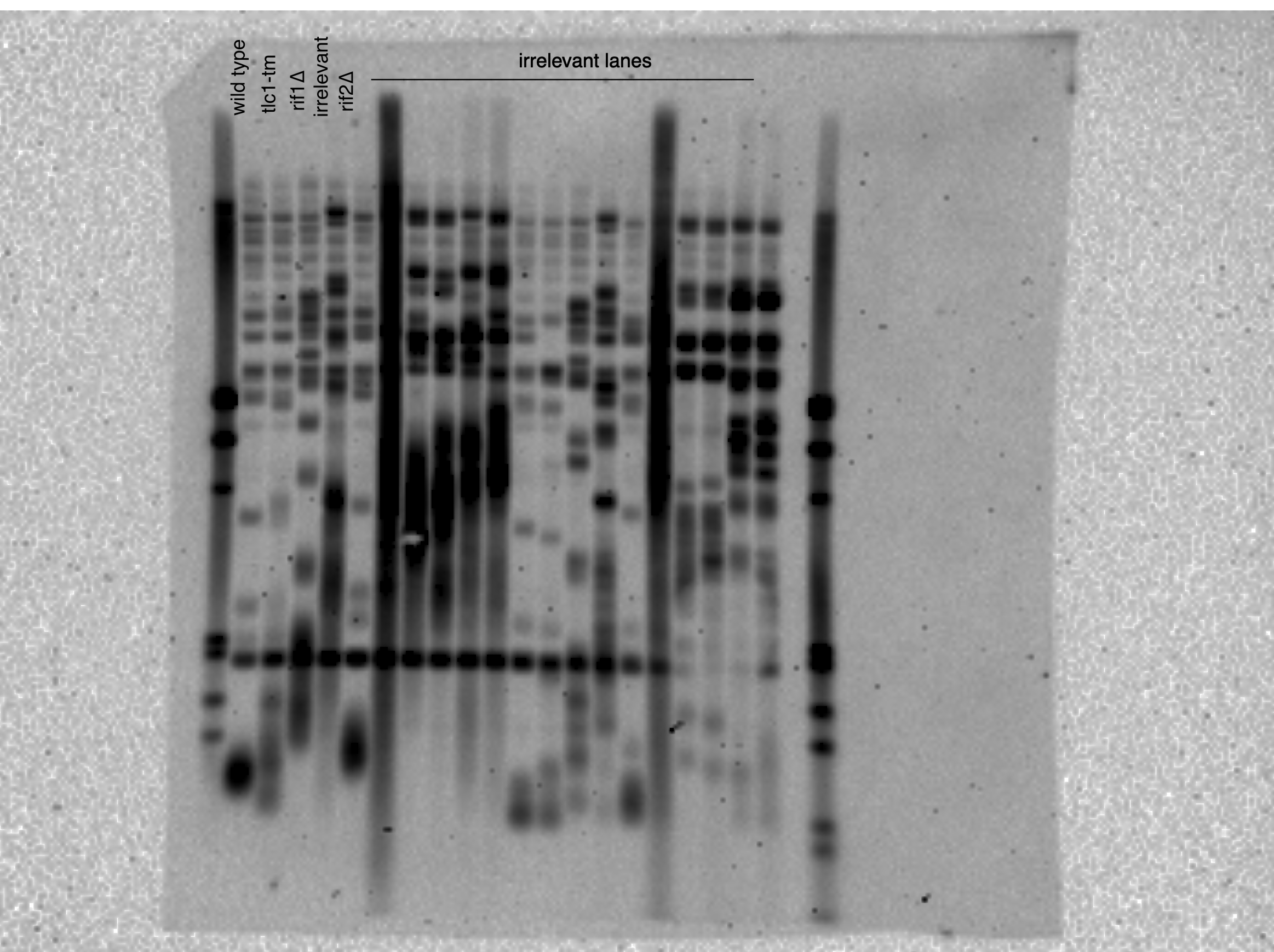

Supplement: Figure 1—source data 2. [file elife-74090-fig1-data2.zip › Fig1-source data2/Fig1B-source data_labeled.tif]

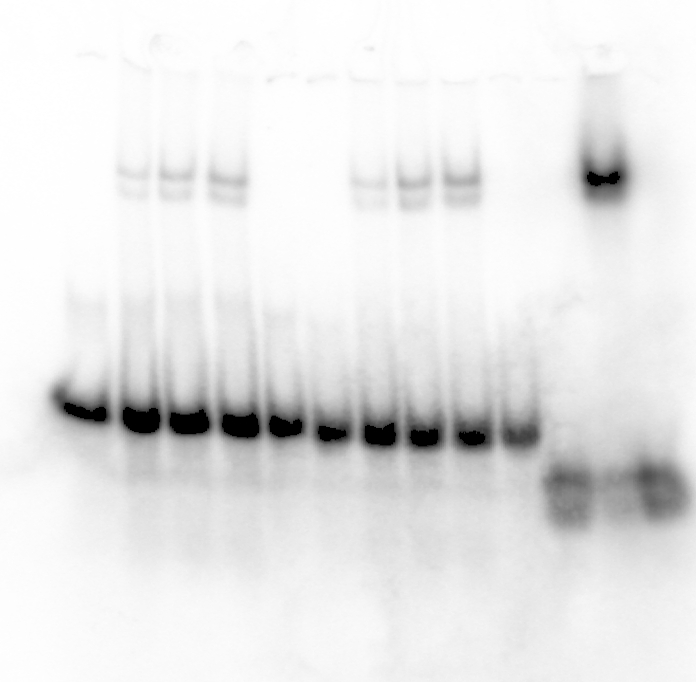

Supplement: Figure 1—source data 2. [file elife-74090-fig1-data2.zip › Fig1-source data2/Fig1E-source data.tiff]

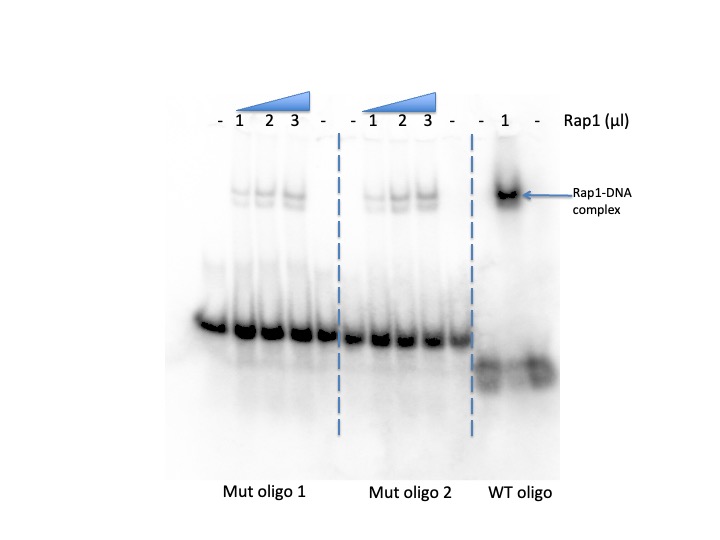

Supplement: Figure 1—source data 2. [file elife-74090-fig1-data2.zip › Fig1-source data2/Fig1E-source data_labeled.jpeg]

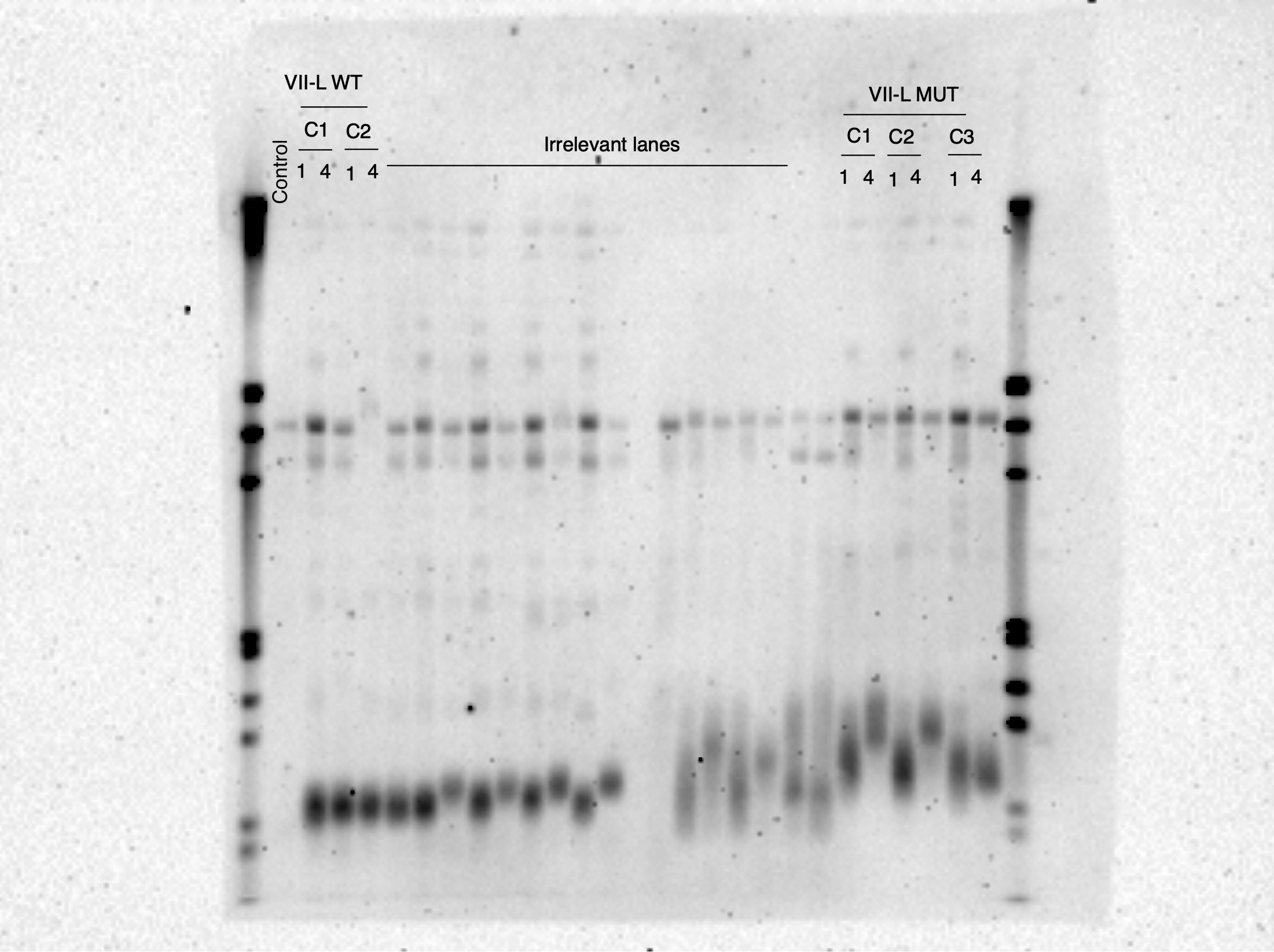

Supplement: Figure 1—source data 2. [file elife-74090-fig1-data2.zip › Fig1-source data2/Fig1C-source data_labeled.tif]

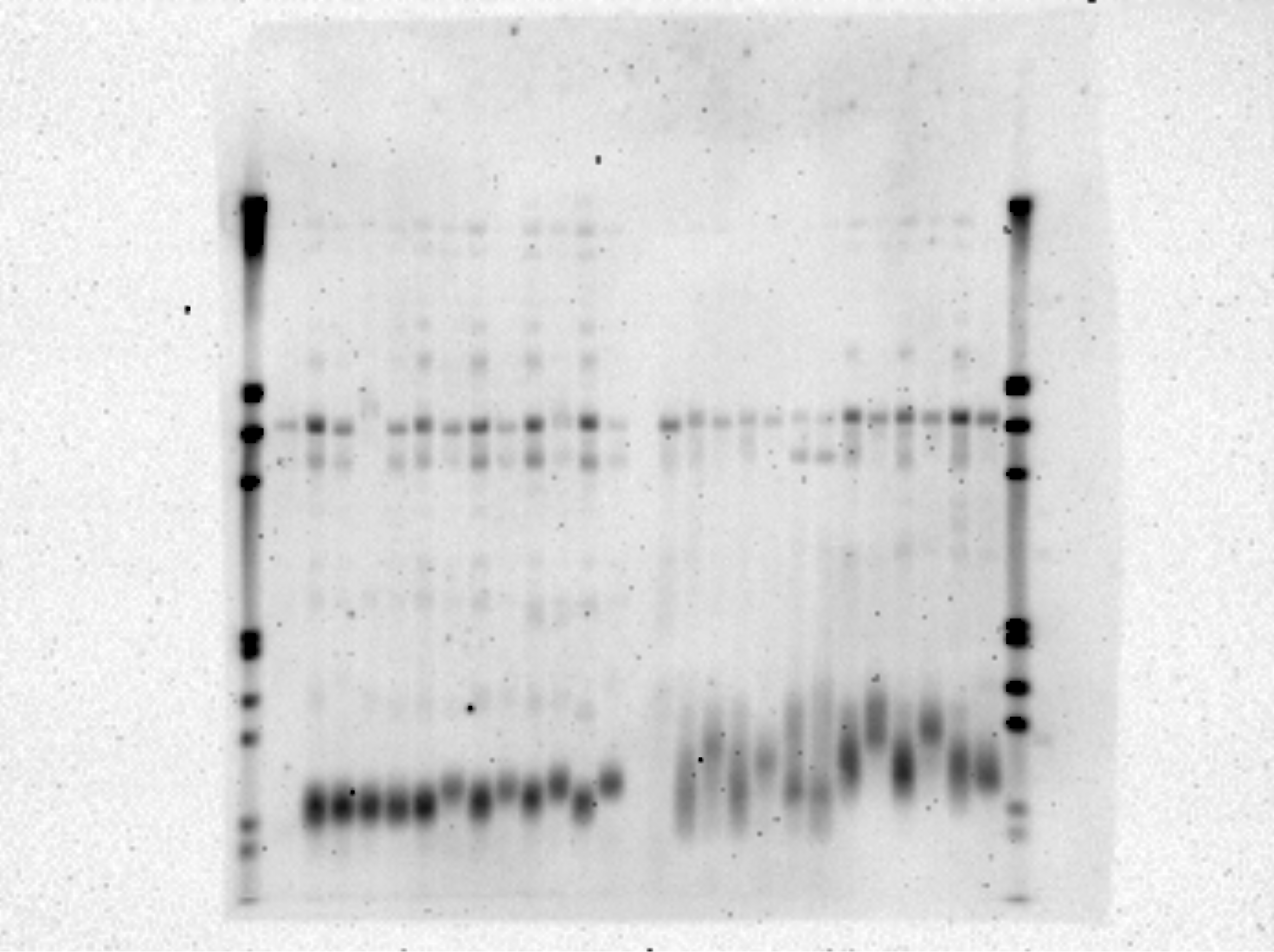

Supplement: Figure 1—source data 2. [file elife-74090-fig1-data2.zip › Fig1-source data2/Fig1C-source data.tif]

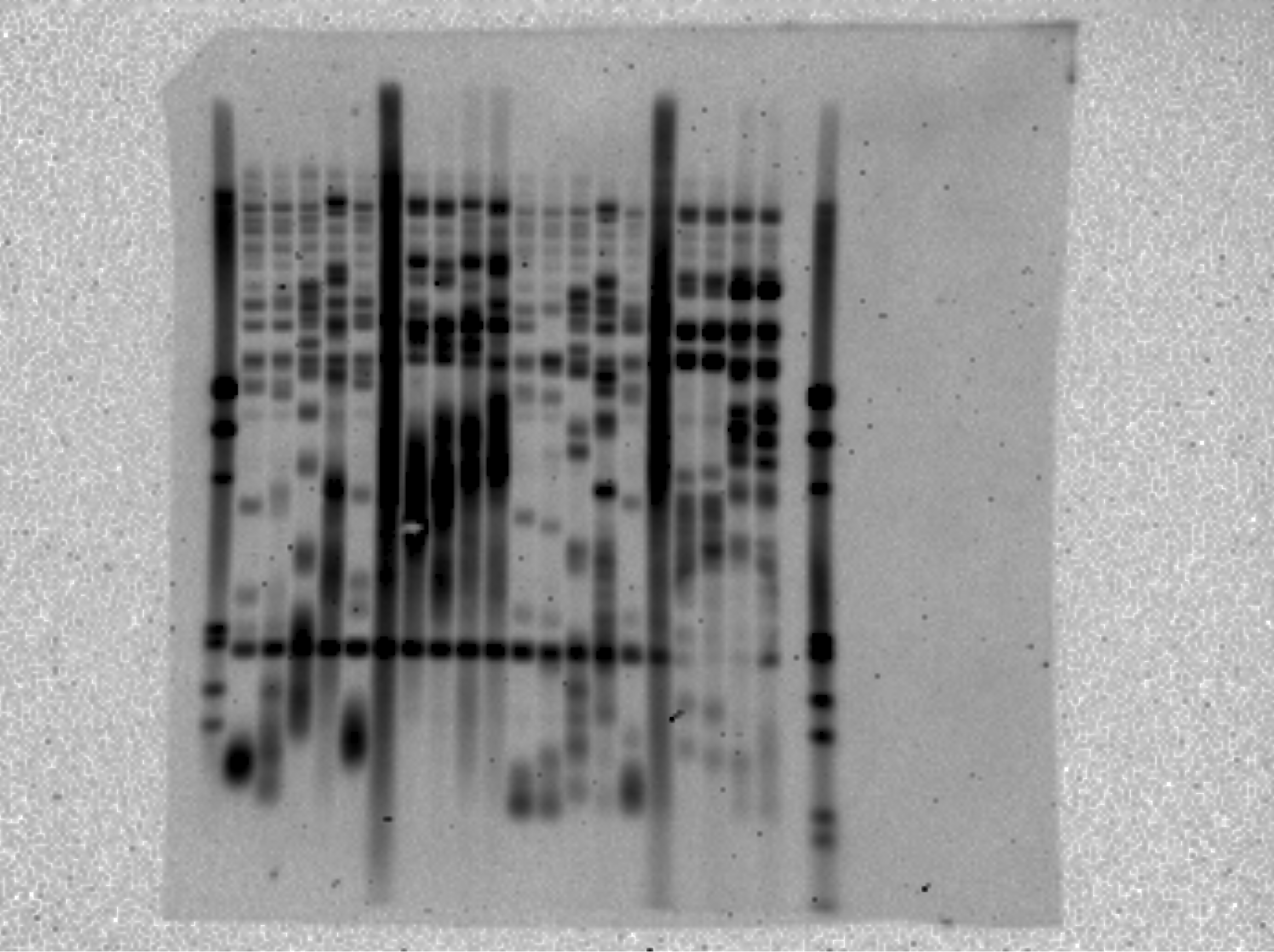

Supplement: Figure 1—source data 2. [file elife-74090-fig1-data2.zip › Fig1-source data2/Fig1B-source data.tif]

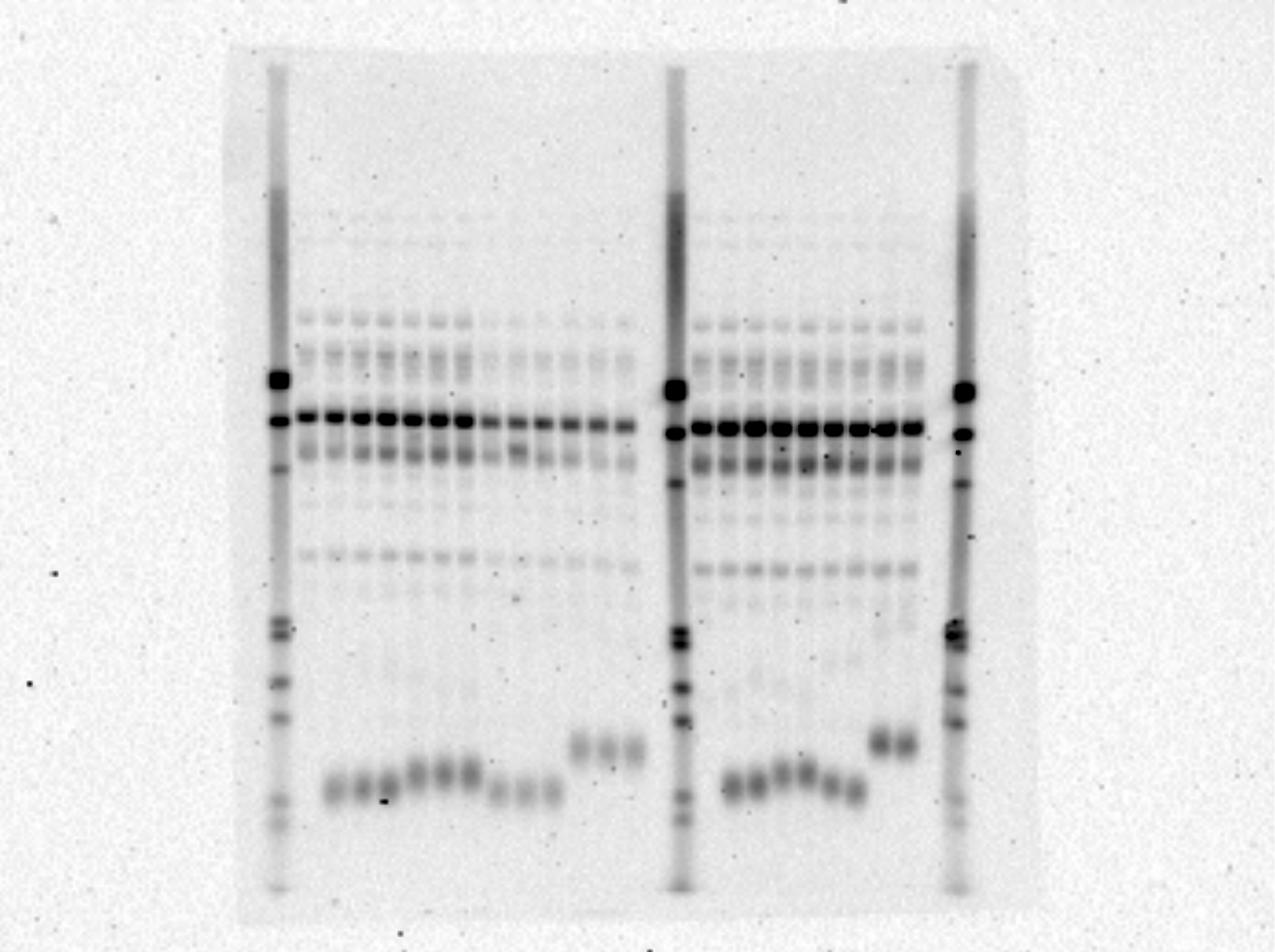

Supplement: Figure 2—source data 1. [file elife-74090-fig2-data1.zip › Fig2-source data1/Fig2A-source data.tif]

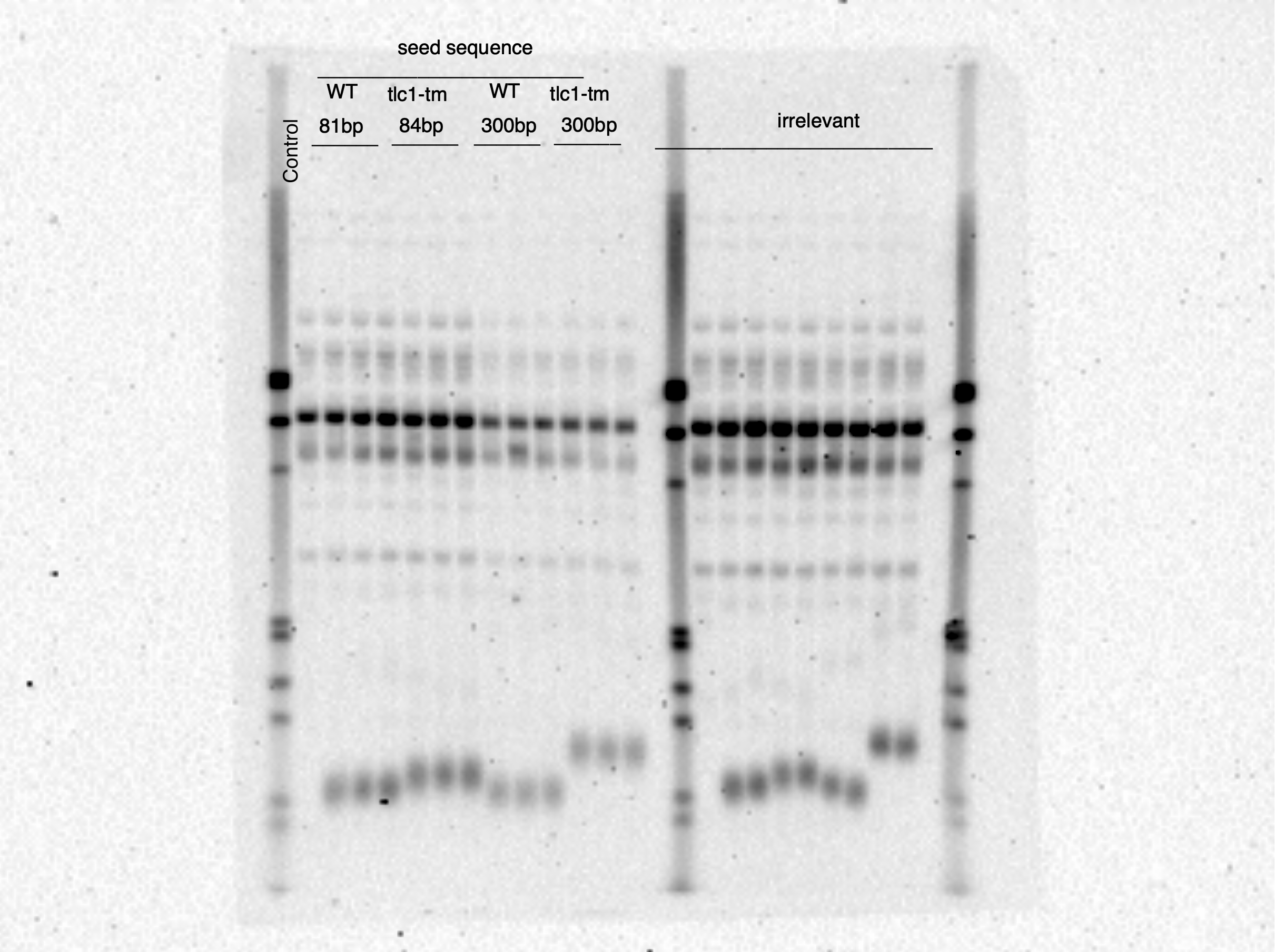

Supplement: Figure 2—source data 1. [file elife-74090-fig2-data1.zip › Fig2-source data1/Fig2A-source data_labeled.tif]

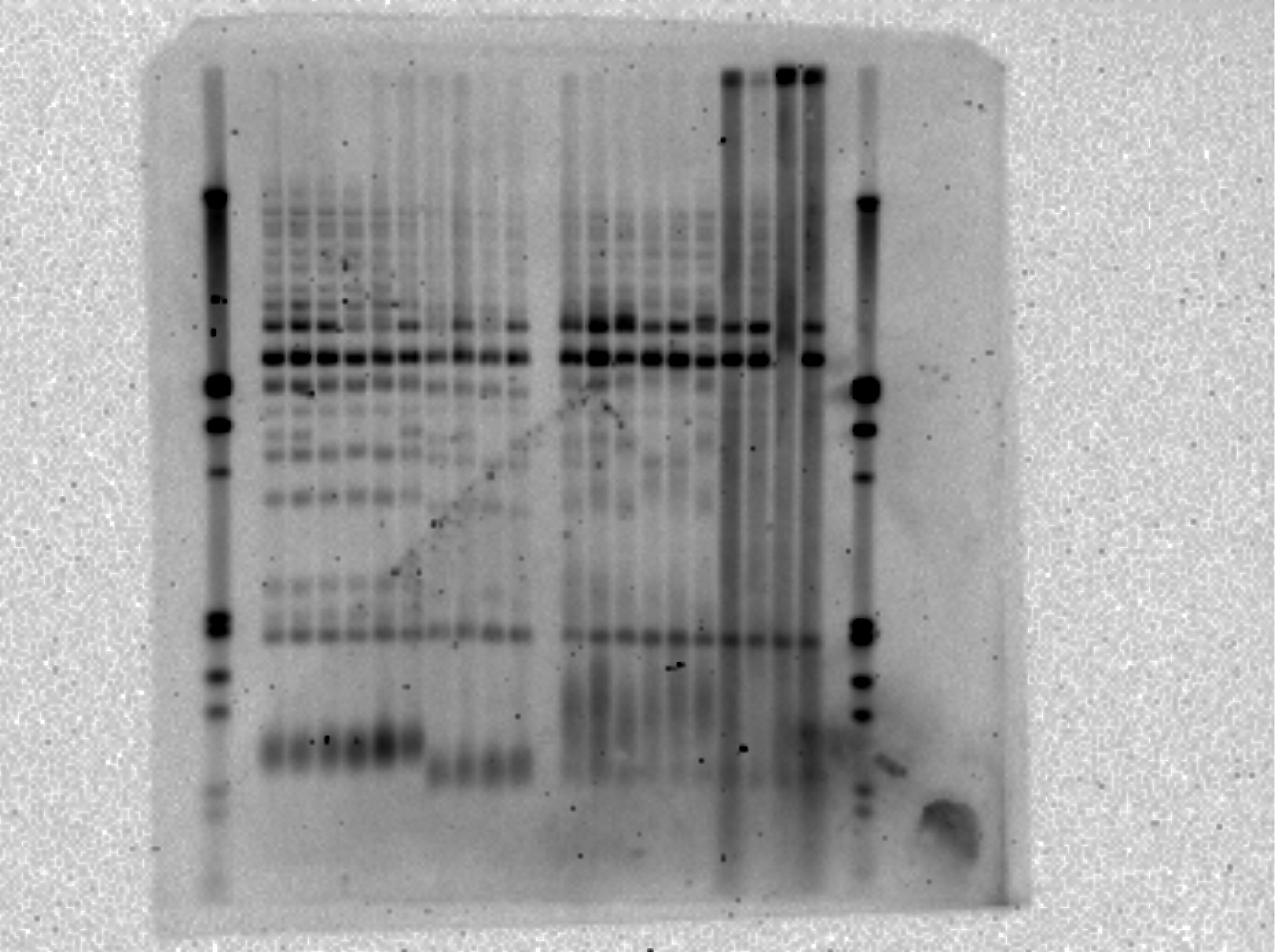

Supplement: Figure 3—source data 1. [file elife-74090-fig3-data1.zip › Fig3-source data1/Fig3B-source data.tif]

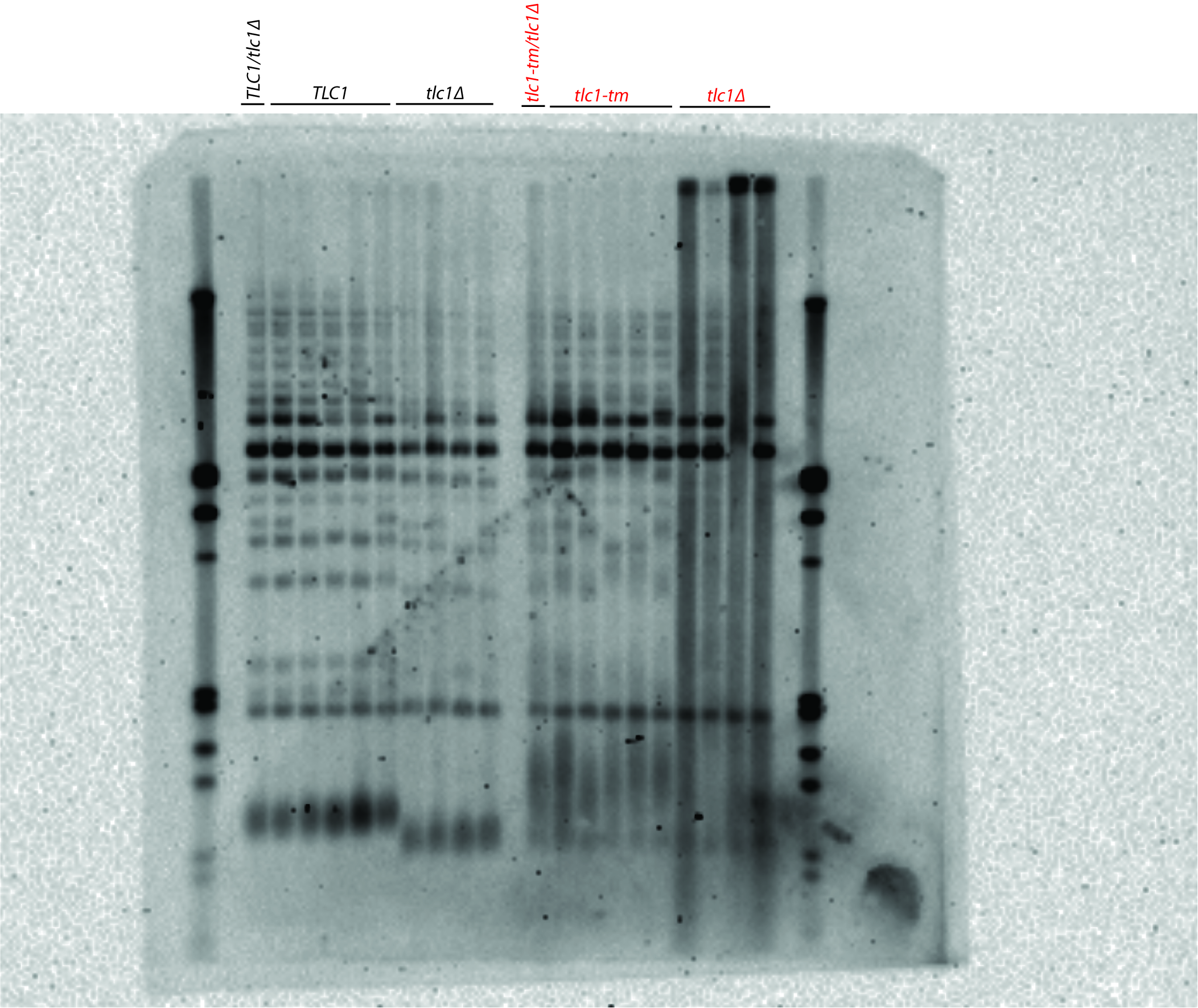

Supplement: Figure 3—source data 1. [file elife-74090-fig3-data1.zip › Fig3-source data1/Fig3B-source data_labeled.tif]

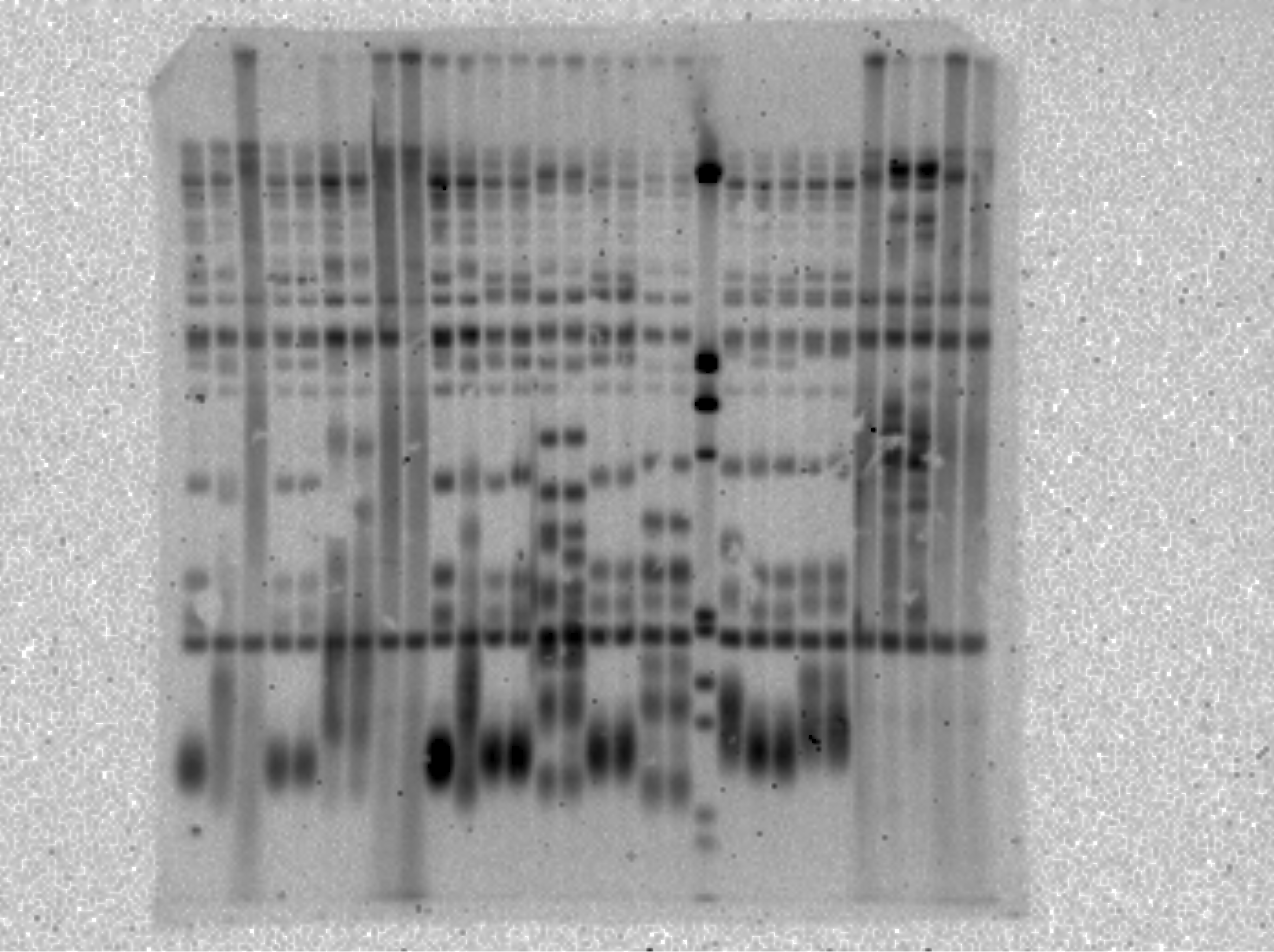

Supplement: Figure 4—source data 2. [file elife-74090-fig4-data2.zip › Fig4-source data2/Fig4-figsupp1-source data.tif]

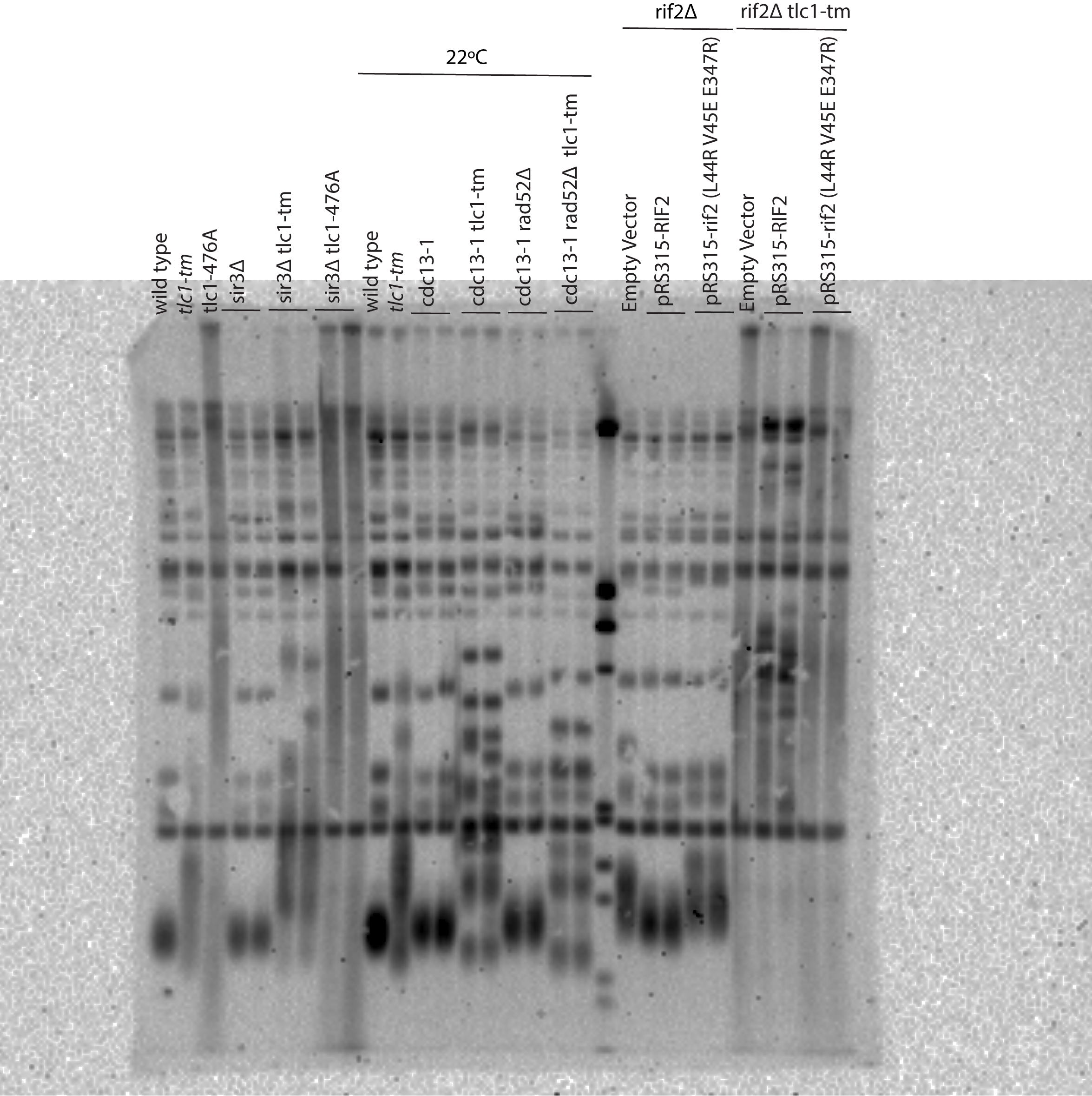

Supplement: Figure 4—source data 2. [file elife-74090-fig4-data2.zip › Fig4-source data2/Fig4-figsupp1-source data_labeled.tif]

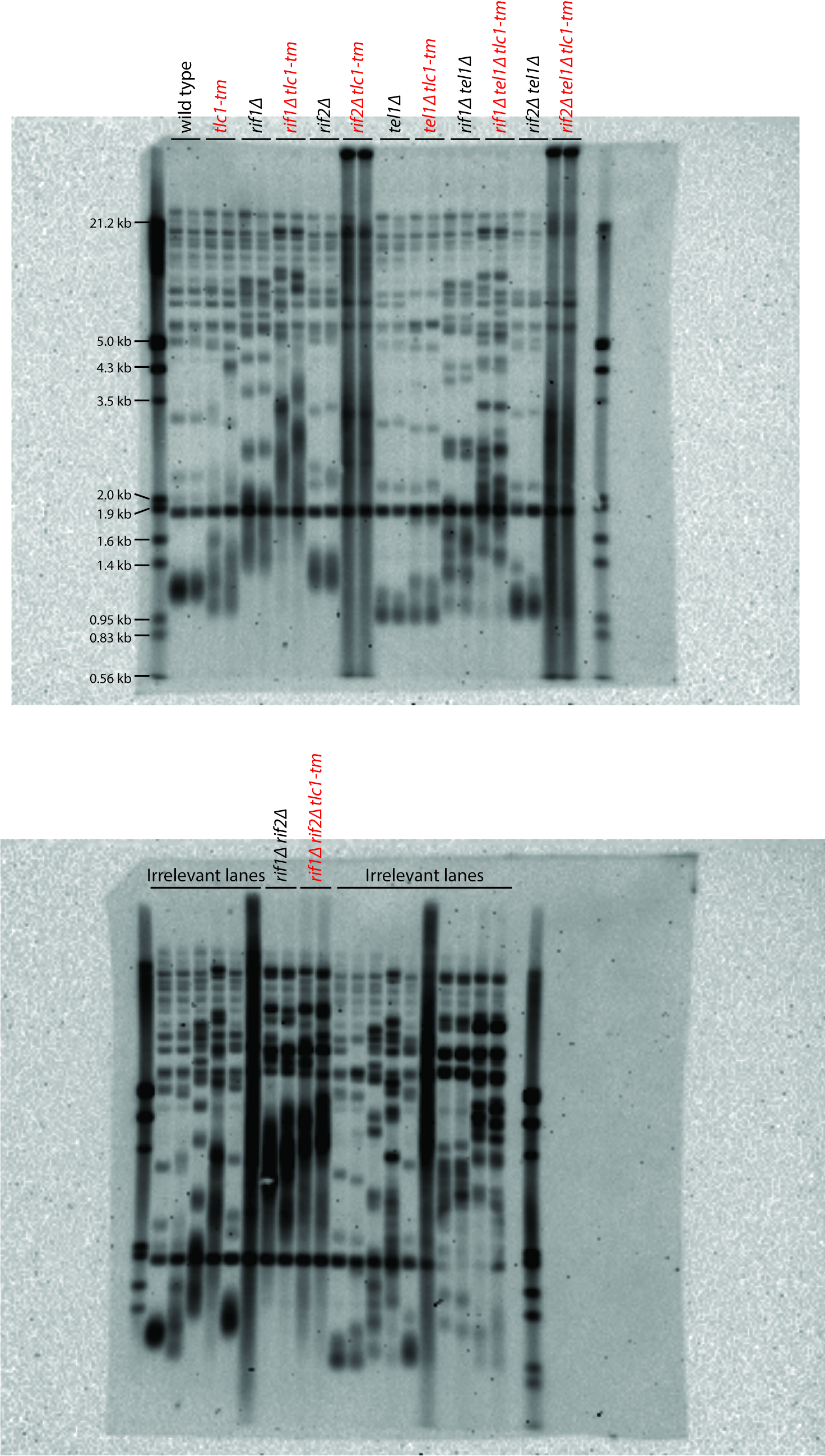

Supplement: Figure 5—source data 1. [file elife-74090-fig5-data1.zip › Fig5-source data1/Fig5B-source data_labeled.tif]

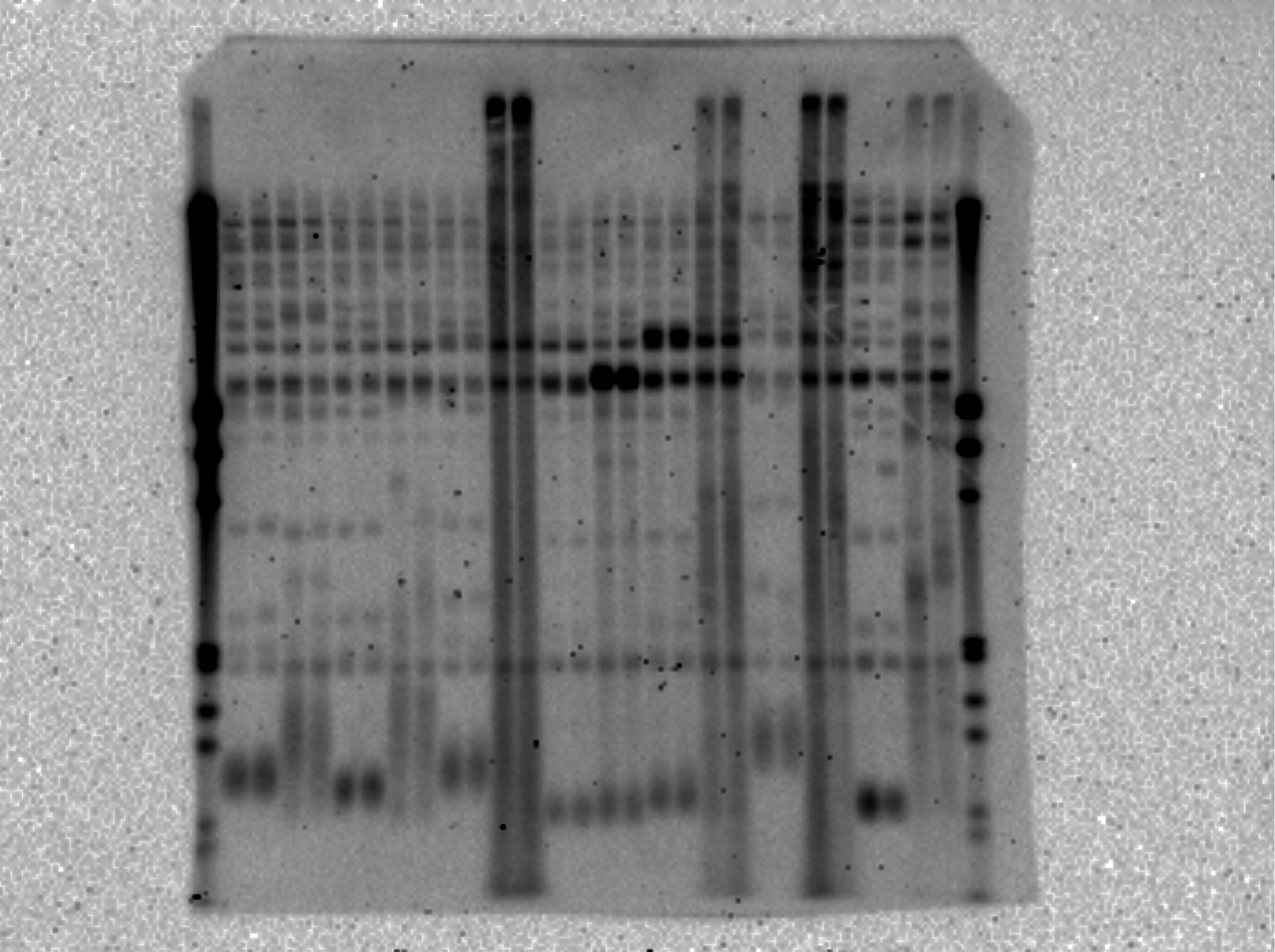

Supplement: Figure 5—source data 1. [file elife-74090-fig5-data1.zip › Fig5-source data1/Fig5A-source data.tif]

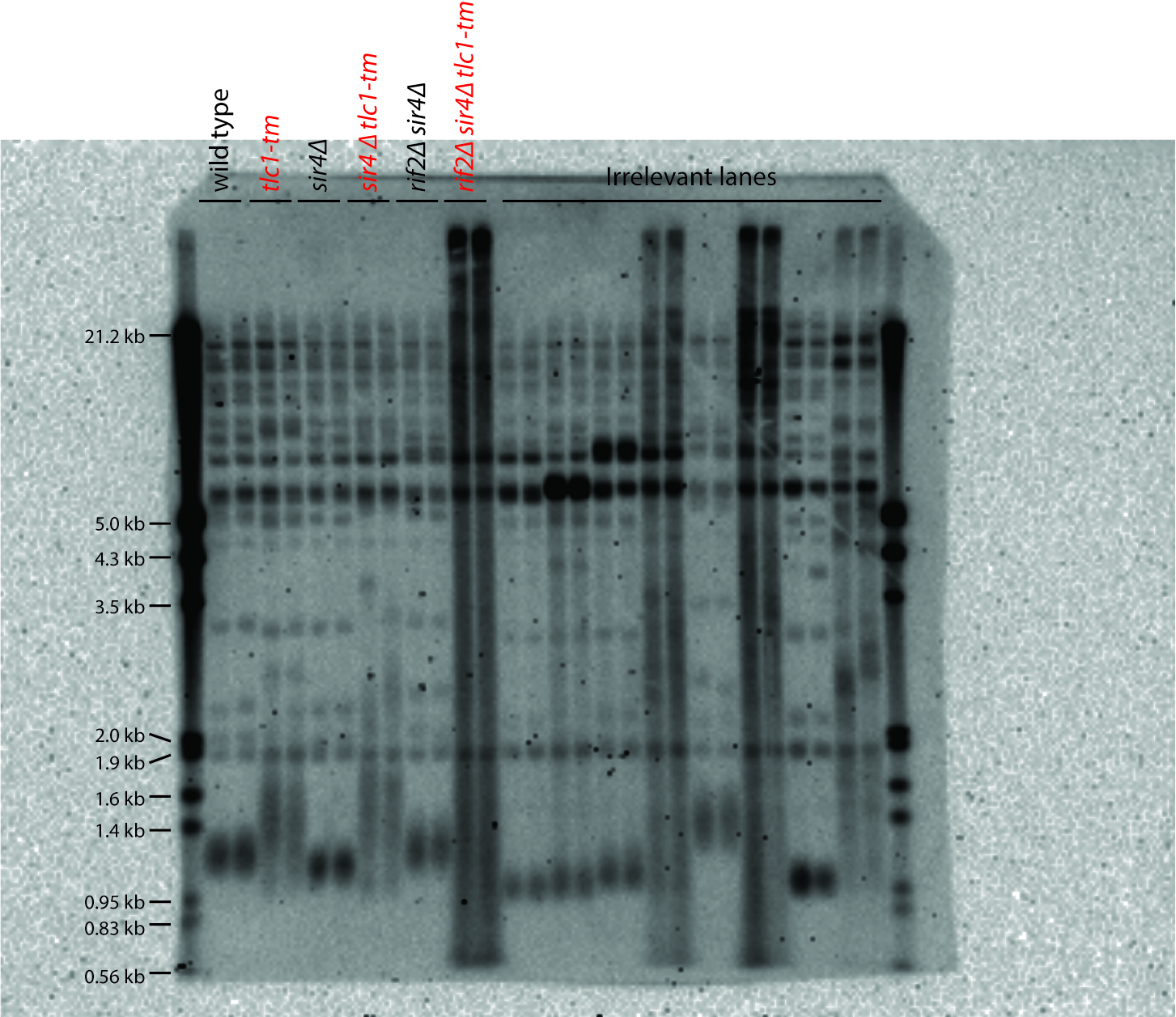

Supplement: Figure 5—source data 1. [file elife-74090-fig5-data1.zip › Fig5-source data1/Fig5A-source data_labeled.tif]

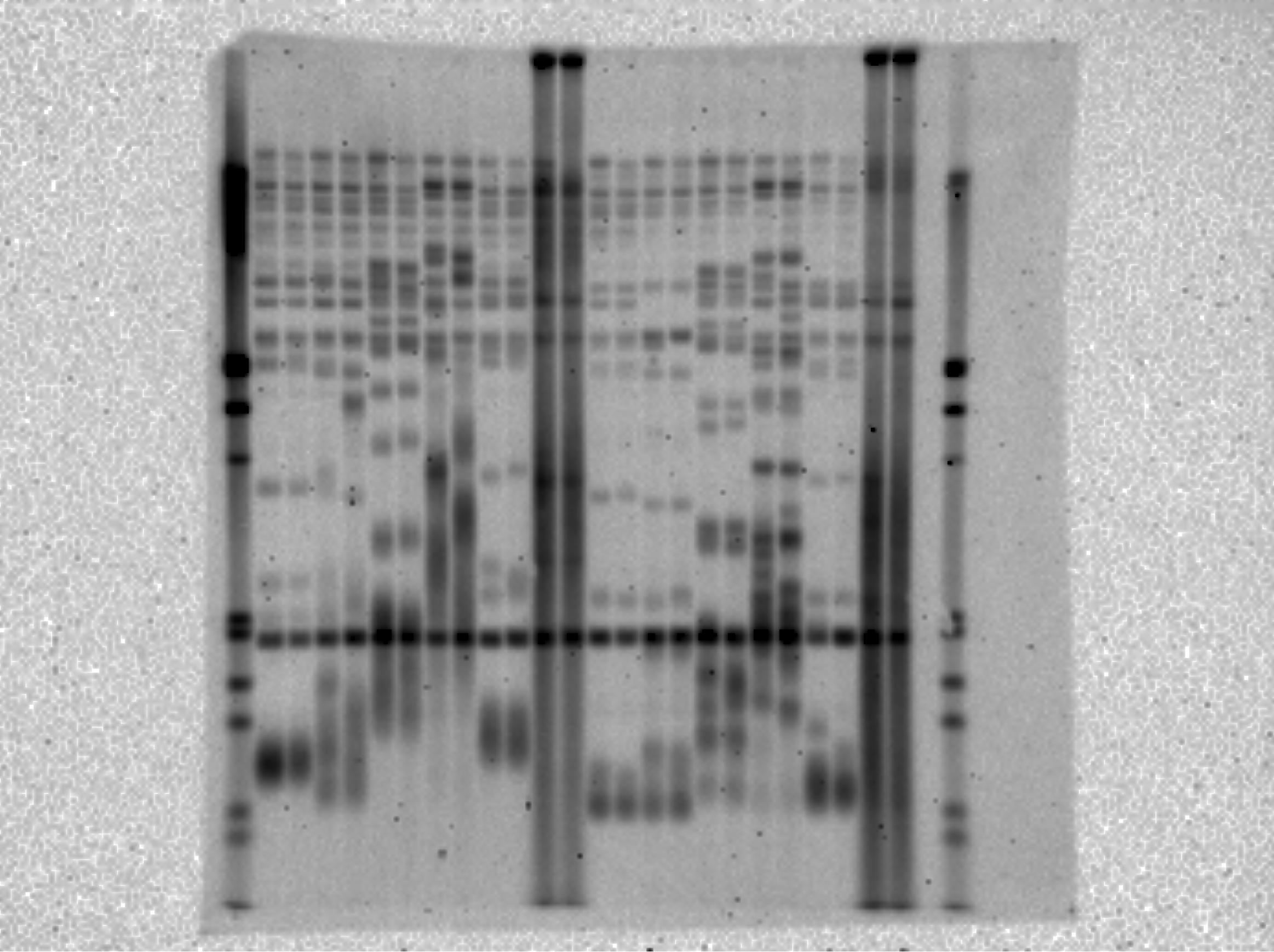

Supplement: Figure 5—source data 1. [file elife-74090-fig5-data1.zip › Fig5-source data1/Fig5Bi-source data.tif]

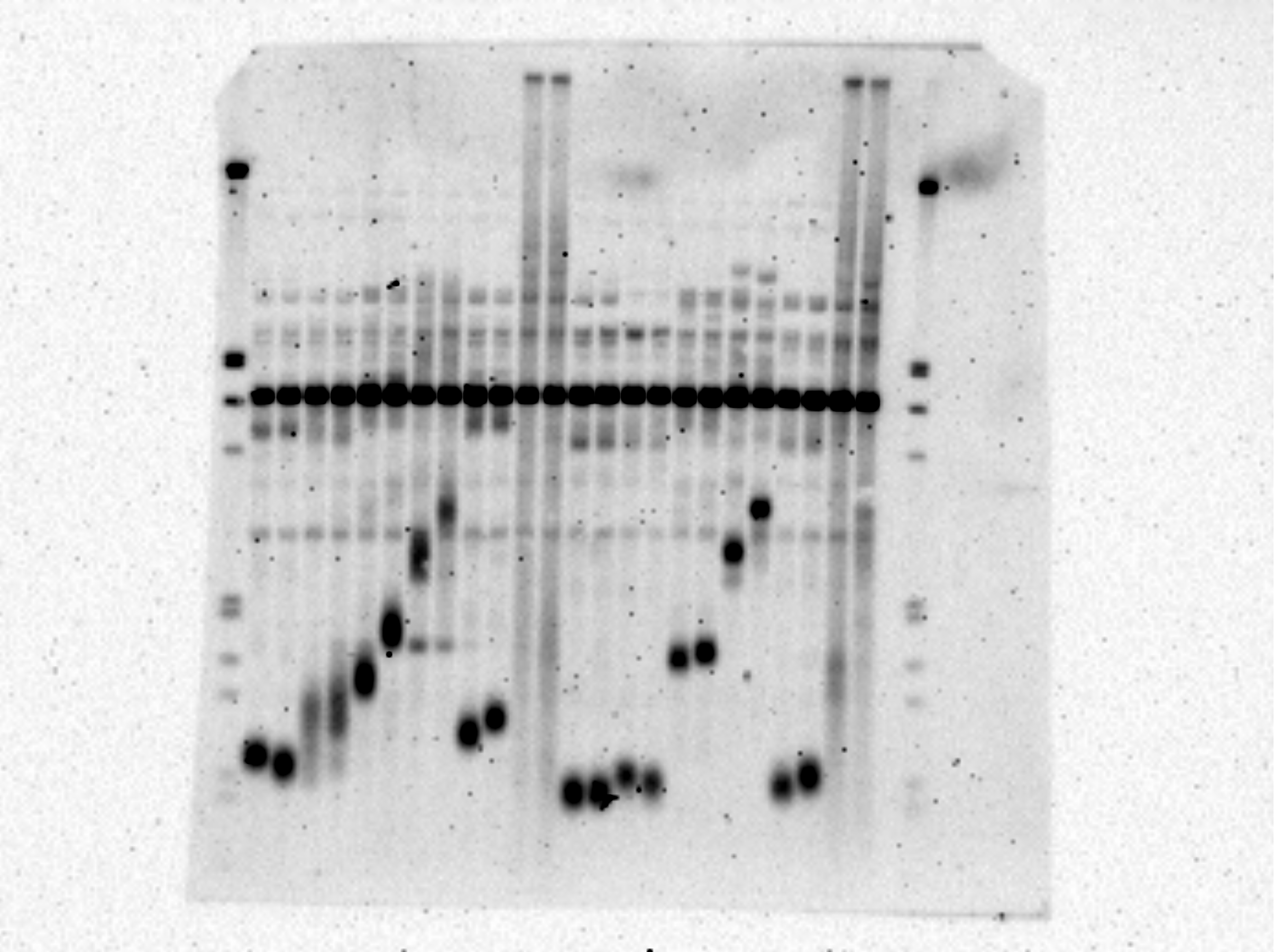

Supplement: Figure 5—source data 1. [file elife-74090-fig5-data1.zip › Fig5-source data1/Fig5-fig supp1-source data.tif]

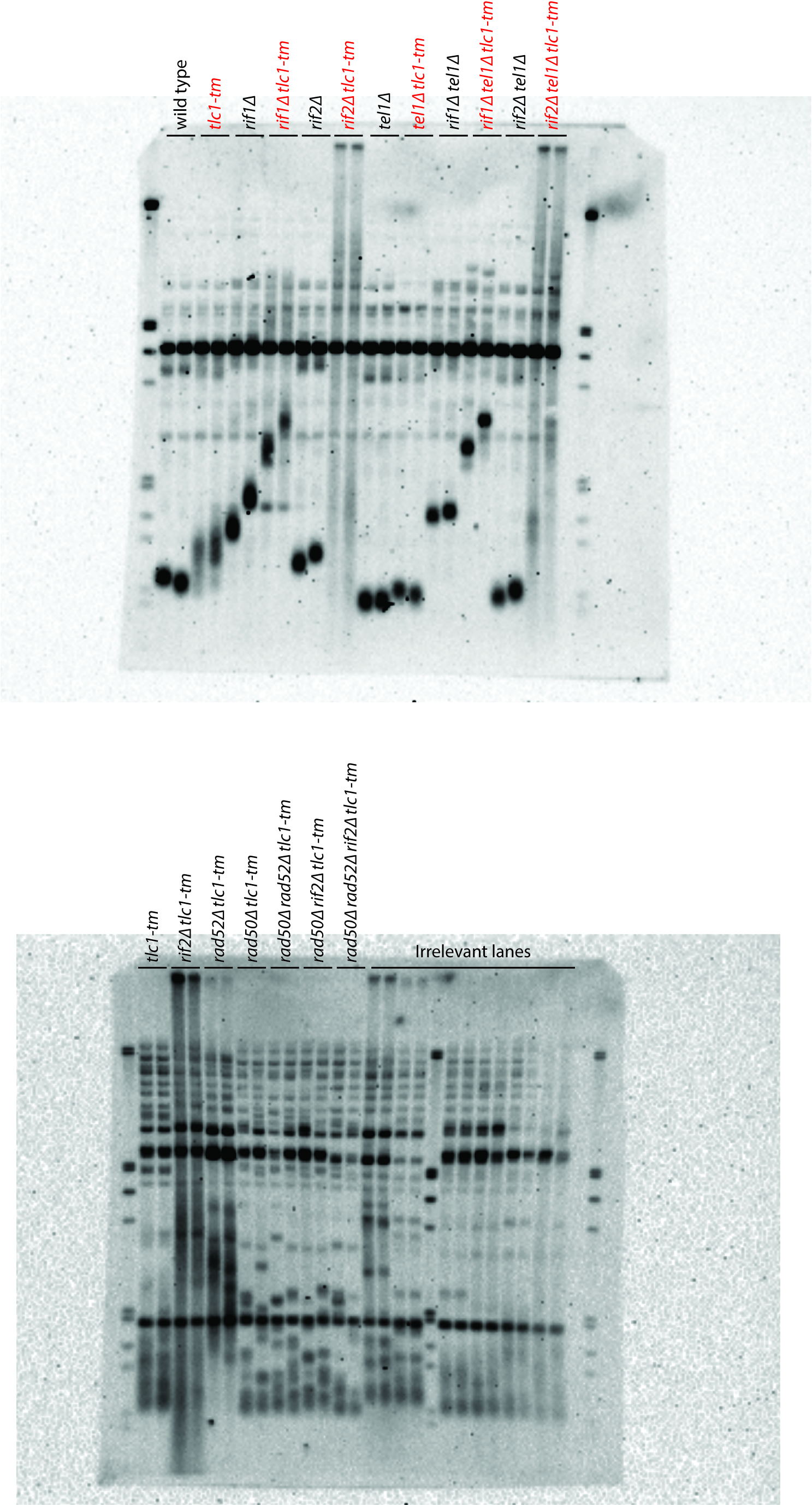

Supplement: Figure 5—source data 1. [file elife-74090-fig5-data1.zip › Fig5-source data1/Fig5-fig supp-source data_labeled.tif]

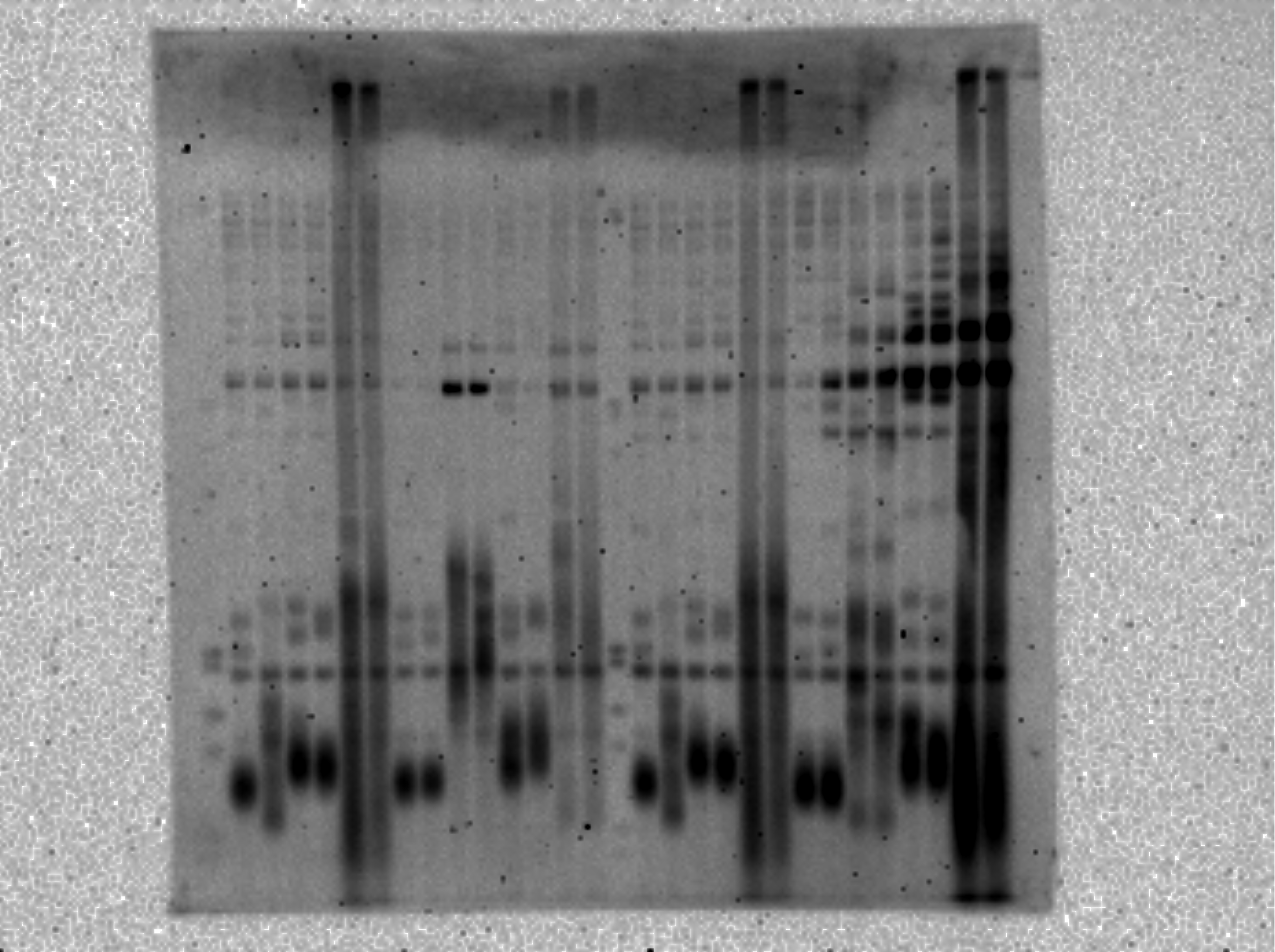

Supplement: Figure 5—source data 1. [file elife-74090-fig5-data1.zip › Fig5-source data1/Fig5C-source data.tif]

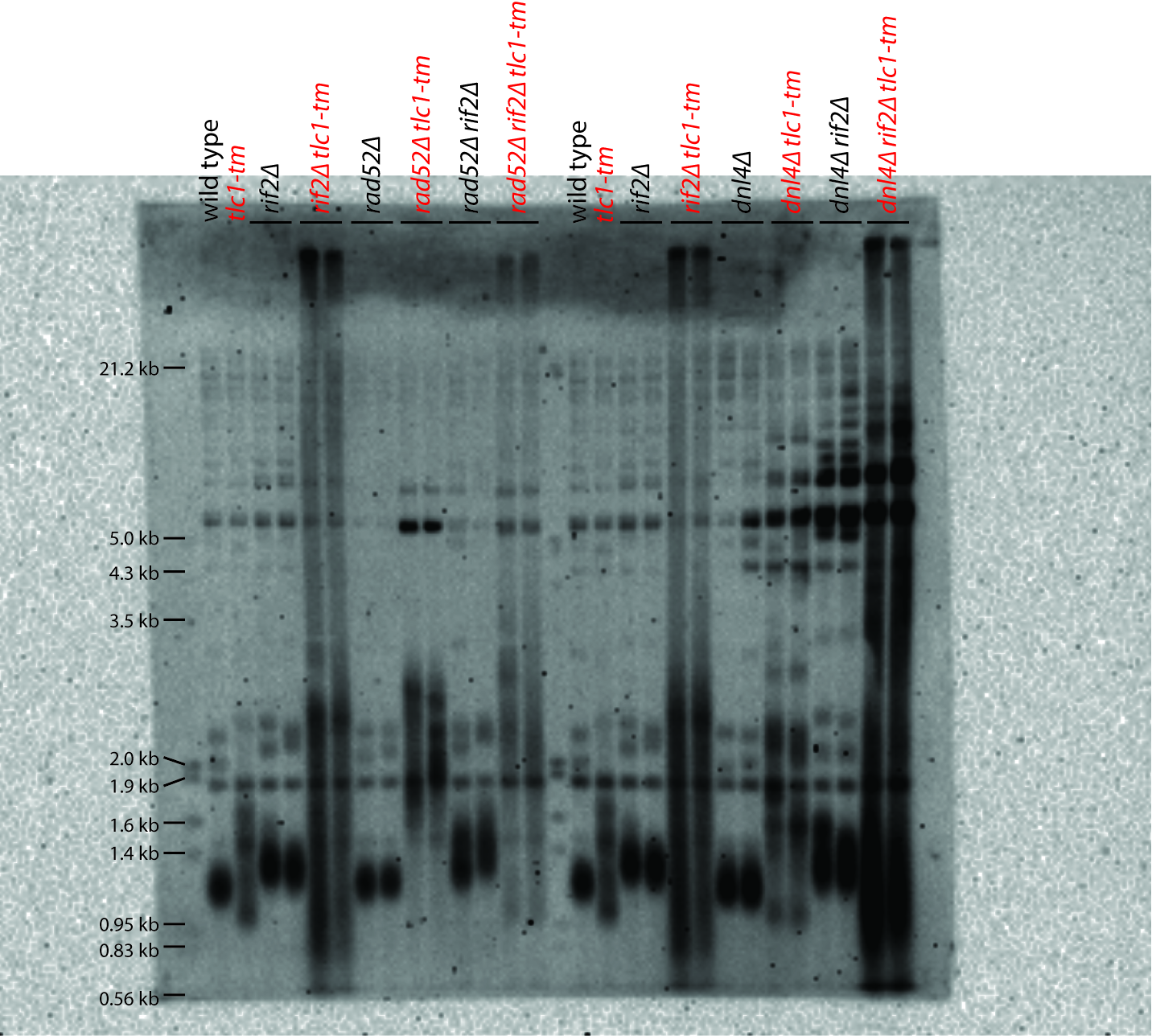

Supplement: Figure 5—source data 1. [file elife-74090-fig5-data1.zip › Fig5-source data1/Fig5C-source data_labeled.tif]

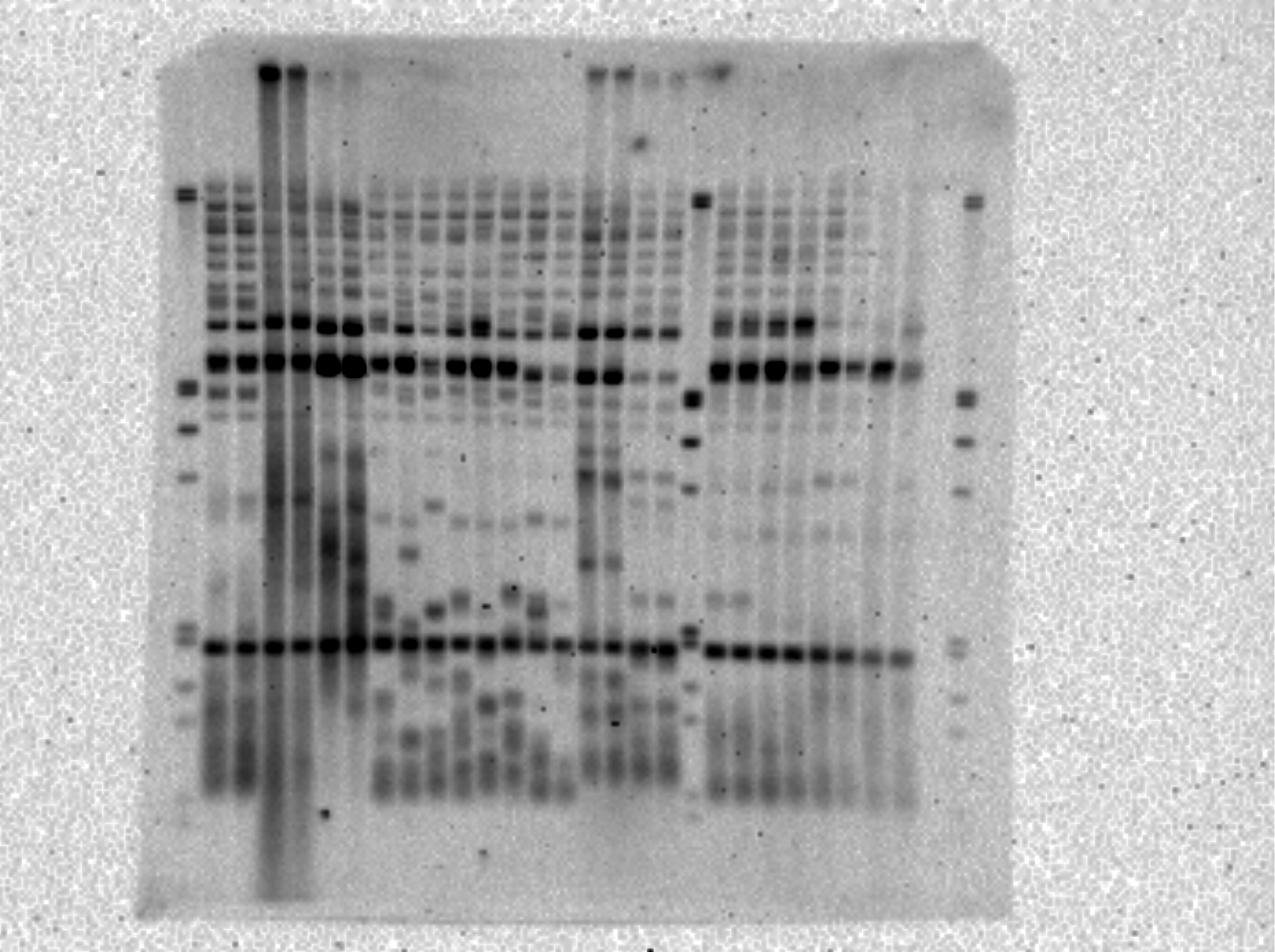

Supplement: Figure 5—source data 1. [file elife-74090-fig5-data1.zip › Fig5-source data1/Fig5-fig supp2-source data.tif]

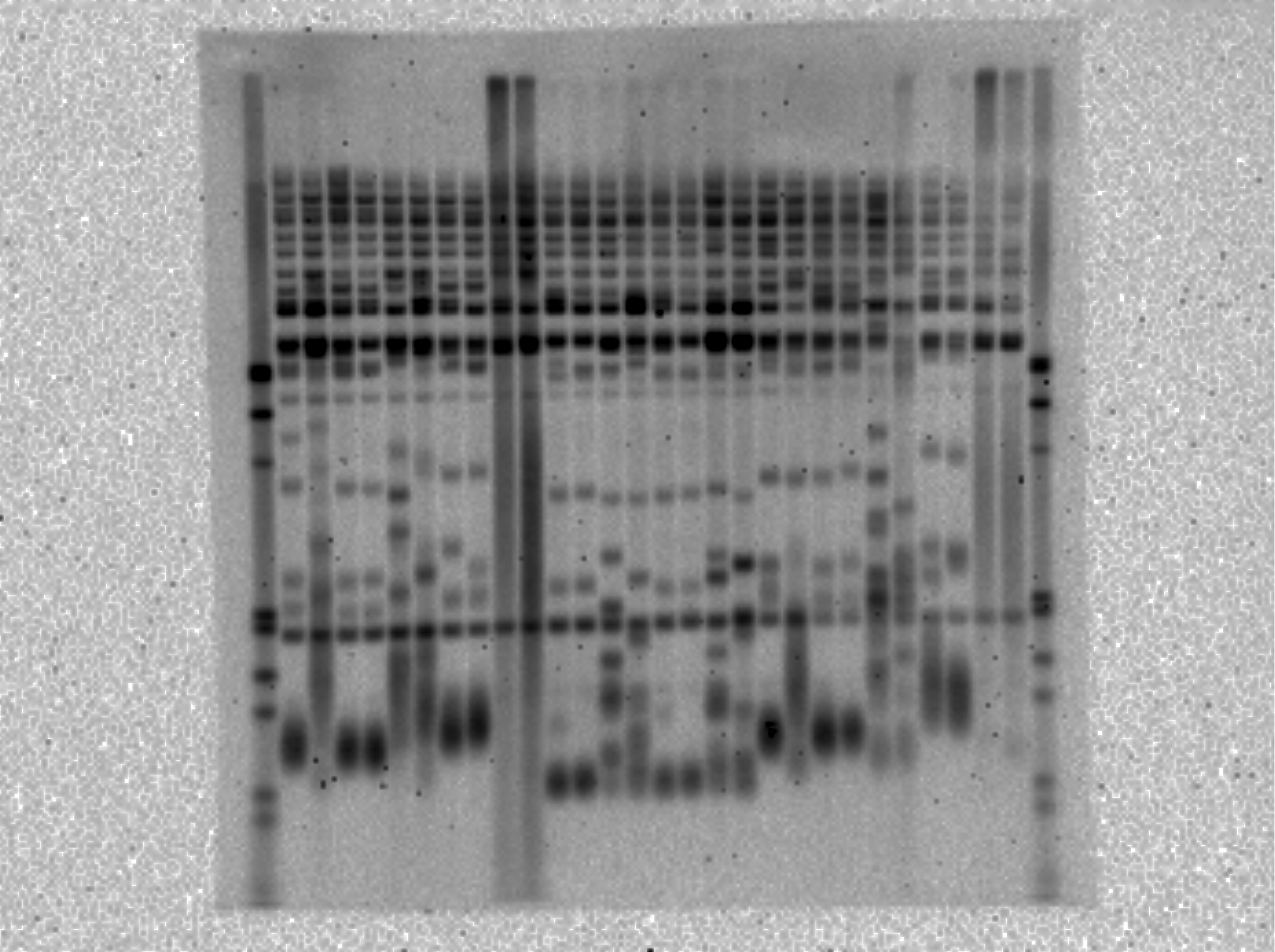

Supplement: Figure 6—source data 2. [file elife-74090-fig6-data2.zip › Fig6-source data2/Fig6B-source data.tif]

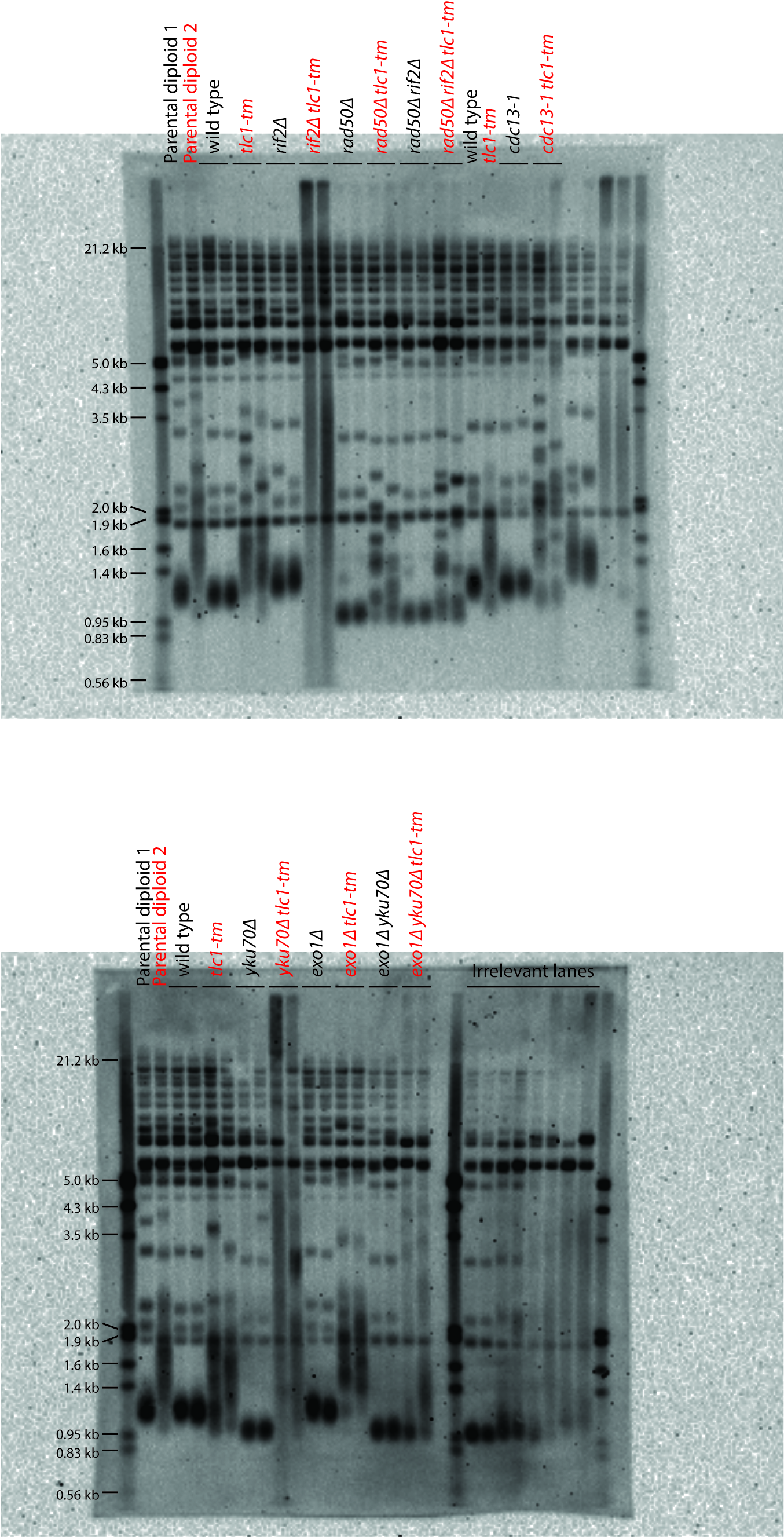

Supplement: Figure 6—source data 2. [file elife-74090-fig6-data2.zip › Fig6-source data2/Fig6BD-source data_labeled.tif]

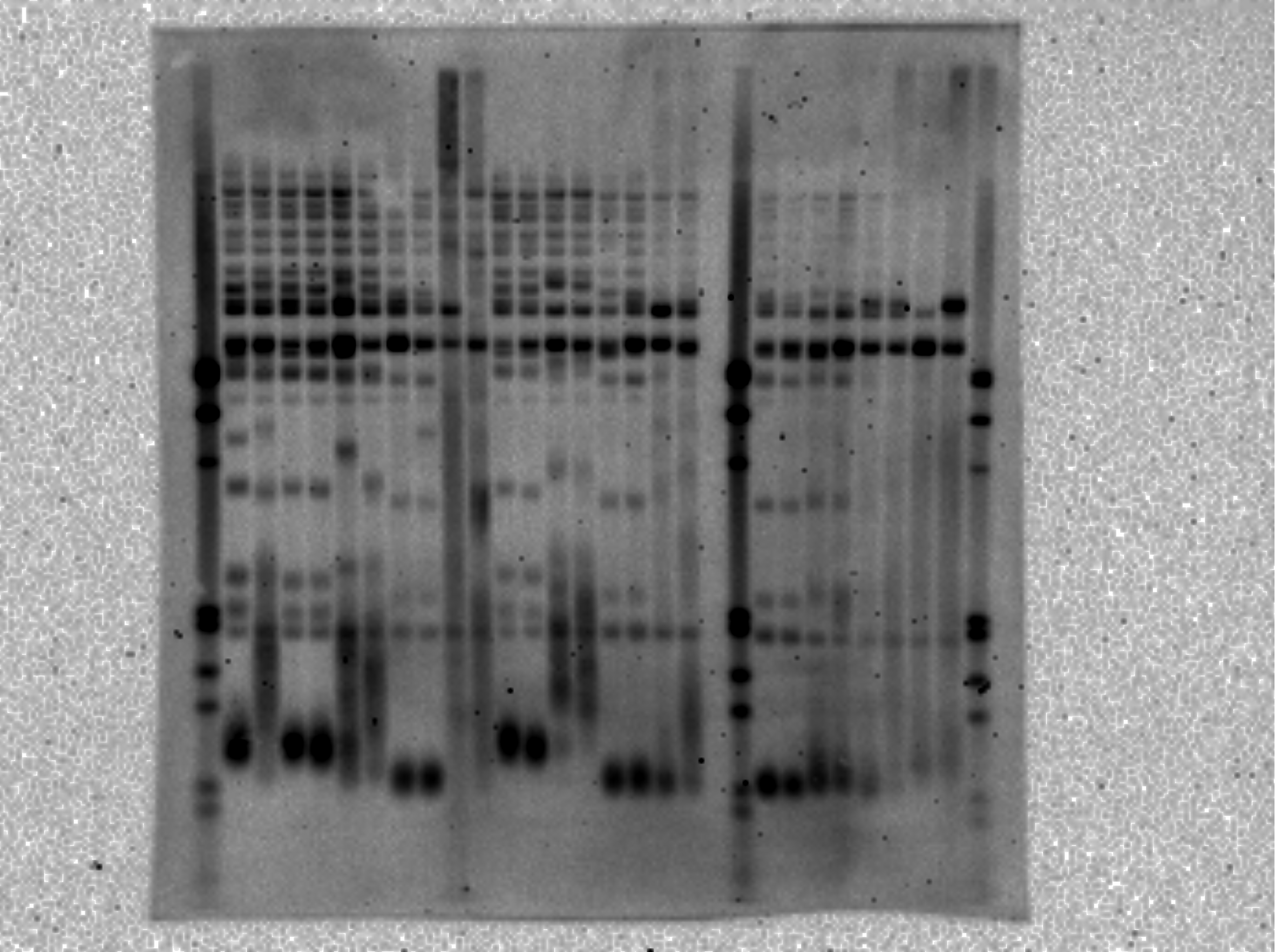

Supplement: Figure 6—source data 2. [file elife-74090-fig6-data2.zip › Fig6-source data2/Fig6D-source data.tif]

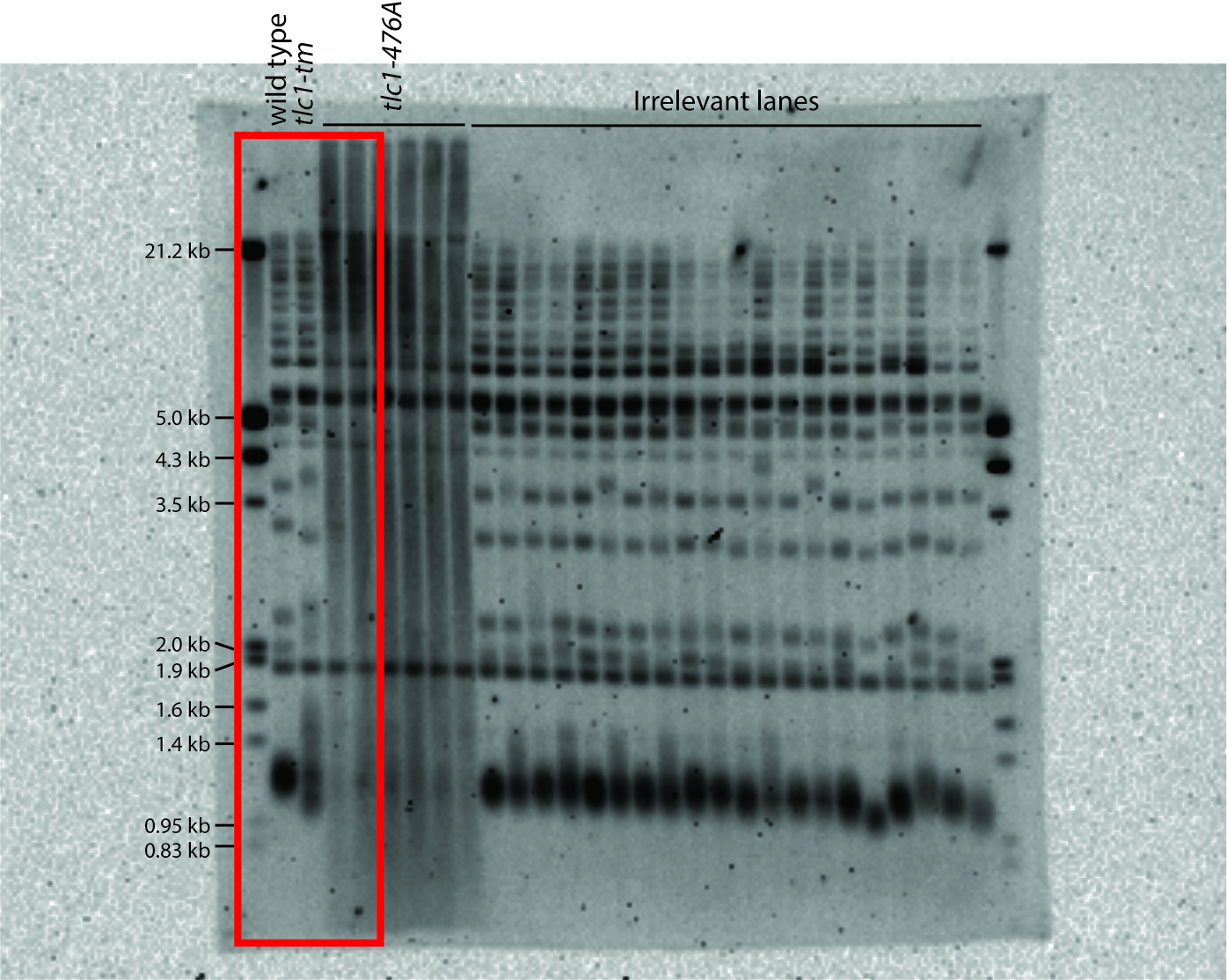

Supplement: Figure 7—source data 1. [file elife-74090-fig7-data1.zip › Fig7-source data1/Fig7A-source data_labeled.tif]

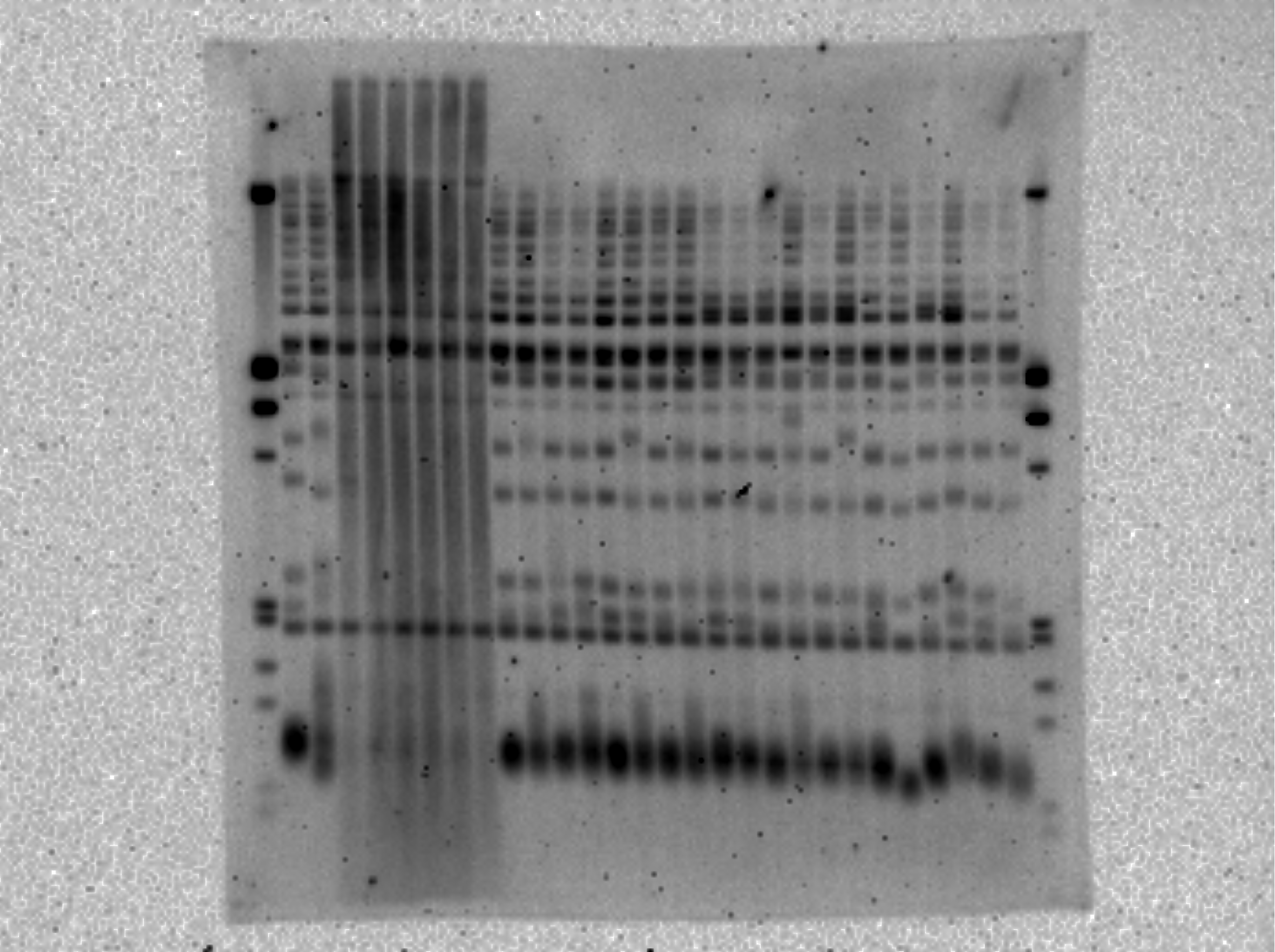

Supplement: Figure 7—source data 1. [file elife-74090-fig7-data1.zip › Fig7-source data1/Fig7A-source data.tif]
